# Quick classification of strong-aroma types of base Baijiu using potentiometric and voltammetric electronic tongue combined with chemometric techniques

**DOI:** 10.3389/fnut.2022.977929

**Published:** 2022-09-12

**Authors:** Ling Ao, Kai Guo, Xinran Dai, Wei Dong, Xiaotao Sun, Baoguo Sun, Jinyuan Sun, Guoying Liu, Anjun Li, Hehe Li, Fuping Zheng

**Affiliations:** ^1^Beijing Laboratory of Food Quality and Safety, Beijing Technology and Business University, Beijing, China; ^2^Key Laboratory of Brewing Molecular Engineering of China Light Industry, School of Light Industry, Beijing, China; ^3^Center for Solid-state Fermentation Engineering of Anhui Province, Bozhou, China

**Keywords:** strong-aroma types of base Baijiu, electronic tongue, principal component analysis, discriminant function analysis, GC-MS

## Abstract

Nowadays, the classification of strong-aroma types of base Baijiu (base SAB) is mainly achieved by human sensory evaluation. However, prolonged tasting brings difficulties for sommeliers in guaranteeing the consistency of results, and may even cause health problems. Herein, an electronic tongue (E-Tongue) combined with a gas chromatography-mass spectrometry (GC-MS) method was successfully developed to grade high-alcoholic base SAB. The E-tongue was capable of identifying base SAB samples into four grades by a discriminant function analysis (DFA) model based on human sensory evaluation results. More importantly, it could effectively and rapidly predict the quality grade of unknown base SAB with an average accuracy up to 95%. The differences of chemical components between base SAB samples were studied by the GC-MS analysis and 52 aroma compounds were identified. The qualitative and quantitative results showed that with the increase of base SAB grade, the varieties and contents of aroma compounds increased. Overall, the comprehensive analysis of E-tongue data and GC-MS results could be in good agreement with human sensory evaluation results, which also proved that the newly developed method has a potential to be a useful alternative to the overall quality grading of base Baijiu.

## Introduction

Baijiu, also called as Chinese liquor or spirit, is one of the most popular alcoholic beverages in China, with the annual production of about 7.156 billion liters in 2021 (1). Baijiu is mainly composed of water, ethanol, and other trace flavor compounds, in which the mass fraction of flavor compounds account for approximately 1–2% of the total mass of Baijiu. Although the proportion of the aforementioned flavor substances in Baijiu is relatively low, the quantitative blending of these flavor substances will play the most important role in the overall aroma. In decades, on the basis of its aroma characteristics, Baijiu are generally classified into twelve categories, such as strong, soy sauce, light, sesame, Chi, complex (Jian), herblike, Feng, rice, Fuyu, Te, and Laobaigan aroma-types (2). With the largest production among them, strong-aroma types of Baijiu (SAB) have gained popularity due to their sensory characterization, such as rich mellow, sweet, and pure, particularly the fragrant after drinking and aftertaste (3).

Based on the characteristics of SAB production, the fresh distillates (base SAB) and the finished product (commercial SAB) are both called SAB (4). In general, during SAB production, freshly distilled base SAB usually has undesirable characteristics and is not preferable for drinking (5). It needs to go through a long aging process, ranging from months to years, to develop a well-balanced “matured” Baijiu (6). Finally, the aforementioned base Baijiu is blended by sommeliers to obtain commercial SAB with standardized flavor and taste. Until now, the classification of base SAB is conventionally graded according to the human sensory evaluation (7). Moreover, coupled with multivariate data analysis (MVDA), the correlation between the chemical profiles and sensory evaluation of samples could be also demonstrated successfully (8). Recently, the contributions of many key aroma compounds to the overall flavor of Baijiu have been identified in this way, such as the “mud-like” aromas in base SAB (9), retronasal “burnt” flavor in soy sauce aroma-types of Baijiu (10), and the sweetness perception of Baijiu (11). However, the method mentioned above is susceptible to environmental impacts and subjective factors, making it difficult to ensure the results during a prolonged tasting in the busy season. More importantly, long-term and abundant base Baijiu tasting does harm to the sommelier because of the high alcohol content (generally at 60–70% alcohol by volume, ABV). Hence, developing a rapid and efficient method for the base SAB classification is of great value and demand.

The electronic tongue (E-tongue) is a kind of human sensory simulating system, which consists of a number of low-selective sensors and uses advanced mathematical procedures for signal processing based on pattern recognition and multivariate data analysis (12). In recent years, E-tongue has been widely used for the analysis of wines, fruit juices, coffee, milk, and beverages, in addition to the detection of trace amounts of impurities or pollutants in waters (13, 14). Moreover, it also has been extensively used for the discrimination of alcoholic beverages by their variety, age, taste, and geographical origin, and it can eliminate panelist bias for taste evaluation of liquor products (15, 16). For instance, an exploratory study was conducted by Schmidtke et al. whose results indicated that bitterness and astringency could be predicted from wines with good precision by using E-tongue and sensory evaluation (17). Legin et al. evaluated 56 Italian wines by recognition and quantitative analysis of E-tongue and concluded that E-tongue was capable of discriminating Barbera d’Asti and Gutturnio wines (18). However, the sensitivity of sensors is easily reduced or lost in high alcohol content.

Due to the high alcohol content of base SAB, the ability of alcohol tolerance of sensors determines whether they are suitable for the analysis of base SAB. Currently, it has been proved by numerous researchers that E-tongue based on inert metal electrodes or modified epoxy-composite sensors could be a good instrument for the distilling spirits analysis (19, 20). Among them, the Smartongue is a kind of E-tongue based on multifrequency large amplitude pulse voltammetry (MLAPV). More importantly, based on a combination of pulse applied relaxation techniques combined with the specific pattern recognition system and multivariate statistical analysis, a lot of signals could be processed more accurately and effectively by MLAPV compared with other sensors (16, 20). In 2007, Tian et al. discriminated six Baijiu samples successfully by using an electronic tongue based on MLAPV coupled with a series of metal electrodes at different frequency segments (21). However, to the best of our knowledge, only a few studies have been reported dealing with the application of E-tongue based on inert metal electrode sensors for the classification of base SAB. Moreover, they simply focused on the differences in statistics without considering the chemical composition.

Therefore, the main objectives of this work were to (i) grade the quality attributes of base SAB by the electronic tongue combined with gas chromatography-mass spectrometry (GC-MS), (ii) establish prediction models by principal component analysis (PCA) or discriminant function analysis (DFA) according to sensory evaluation results, and (iii) better elucidate the differences of aroma compounds from each grade of base SAB using the GC-MS analysis and compared with the national standard of GB/T 10781.1-2021. These results will be useful for the quick quality grading of SAB from the Baijiu industry.

## Materials and methods

### Chemicals

Authentic standards, including twenty-seven kinds of esters (ethyl acetate, ethyl butanoate, ethyl pentanoate, ethyl 4-methylpentanoate, ethyl hexanoate, hexyl acetate, propyl hexanoate, ethyl lactate, ethyl heptanoate, butyl hexanoate, ethyl octanoate, isopentyl hexanoate, hexyl hexanoate, ethyl decanoate, isoamyl octanoate, ethyl benzoate, diethyl butanedioate, ethyl phenylacetate, hexyl octanoate, ethyl dodecanoate, ethyl 3-phenylpropionate, ethyl tetradecanoate, ethyl hexadecanoate, ethyl octadecanoate, ethyl oleate, ethyl linoleate, and ethyl linolenate), twelve types of alcohols (1-propanol, 1-butanol, 2-butanol, 2-methylpropanol, 1-pentanol, 2-pentanol, 2-methylbutanol, 3-methylbutanol, 1-hexanol, 1-octanol, 2,3-butanediol, and phenylethyl alcohol), nine kinds of acids (acetic acid, 2-methylpropanoic acid, butanoic acid, 3-methylbutanoic acid, pentanoic acid, 4-methylpentanoic acid, hexanoic acid, heptanoic acid, and octanoic acid), three kinds of phenols (4-methylguaiacol, 4-ethylguaiacol, and 4-methylphenol), and one kind of acetal (diethyl acetal), were all purchased from Sigma-Aldrich (Beijing, China). Compounds, such as 4-octanol (internal standard, IS1), 4-hydroxy-2-butanone (IS2), n-pentyl acetate (IS3), and 2-ethylbutyric acid (IS4), used as internal standards in this study, were purchased from Sigma-Aldrich (Beijing, China). A C5-C30 n-alkane mixture (Sigma-Aldrich, Beijing, China) was employed for the determination of linear retention indices (RIs). Sodium chloride, anhydrous sodium sulfate, dichloromethane, kalium chloratum, and absolute ethanol were purchased from Sinopharm Chemical Reagent Co., Ltd. (Beijing, China). All the chemicals used above were of analytical reagent grade, with at least 97% purity.

### Sampling and sample preparation

#### Sampling

A total of 140 base SAB samples from 140 different pits were obtained from Anhui Gujing Distillery Co., Ltd., (Anhui, China), with alcohol content ranging from 55 to 70% ABV. All samples (125 ml from each bottle) were stored at 4°C until analysis.

#### Sample preparation of base SAB for liquid-liquid extraction

According to the method reported by Zheng, and associated with some modifications (22), a total of 25 ml of base SAB sample was diluted to 10% ABV with Milli-Q water (Millipore, Bedford, MA, United States). Before being used, the water was boiled for 5 min, and then cooled to 20°C in a 1.0 L flask. The diluted base SAB sample was saturated with NaCl, and extracted 3 times with freshly distilled dichloromethane (50.0 ml each time). The dichloromethane extracts (about 150.0 ml) were dried with plenty of anhydrous Na_2_SO_4_ overnight, concentrated to a final volume of 1.0 ml under a gentle stream of nitrogen, and then the concentrated extracts were stored at −20°C before GC-MS analysis.

### Sensory evaluation

Each base SAB sample (20 ml) was subjected to sensory descriptive judgment by the sommeliers at 20°C after it was poured into a glass cup. The procedure was conducted in a sensory laboratory following the national standard of GB/T 10345-2007 (23). There were eight panelists (4 men and 4 women, composed of a team, including 4 junior sommeliers, 2 intermediate sommeliers, 2 senior sommeliers, and age range 25–40 years) participating in the sensory evaluation session with a weighted score in this research. The weights of junior sommeliers, intermediate sommeliers, and senior sommeliers were 0.8, 1.0, and 1.2, respectively. The panelists were required to rinse their mouth thoroughly with purified water and rest for 1.0 min at least between two-samples-tasting and rest 10.0 min or more per four-samples-tasting. Each sample, randomly marked with a three-digit number, was presented randomly.

### Smartongue analysis

In total, 140 target base SAB samples were analyzed by Smartongue (RuiFen International Trading Co., Ltd., Shanghai, China). The sensor of Smartongue should be preheated successively with 20.0 ml of 0.01 mol/L KCl solution and 20.0 ml SAB samples after each start of Smartongue. The programmed parameters of Smartongue were set as follows: the step voltage was set at 0.2 V, and 6 sensors were all chosen at 10^–4^ sensitivity. Each base SAB (20.0 ml) was subjected to test by the Smartongue at 20°C in a glass cup. Between each measurement, the sensors were rinsed with 25.0 ml of deionized water. Three replicate measurements were conducted for each base SAB sample.

### GC-MS analysis

#### Qualitative analysis

The aroma compounds in the base SAB samples were detected by both direct injection and liquid–liquid extraction coupled with GC-MS method (3). The GC-MS analysis of base SAB samples was performed on an Agilent 7890 gas chromatograph equipped with an Agilent 5977A mass-selective detector (MSD) and a DB-WAX column (60 m × 0.25 mm i.d., 0.25 μm film thickness, J&W Scientific). The column carrier gas was helium at a constant flow rate of 1.0 ml/min and the direct injection volume was 1.0 μl with a split ratio of 5:1. The injector temperature was set at 250°C. The oven temperature was held at 40°C for 0.5 min, then programmed to 50°C at a rate of 10°C/min and held for 8 min and then, programmed to 70°C at a rate of 3°C/min and held for 5 min afterward. Next, it was programmed to 187°C at a rate of 3°C/min and held for 1 min, and finally programmed to 230°C at a rate of 5°C/min and held for 4 min. The temperature of the mass-selective detector transfer line was kept at 240°C. Mass spectra in the electron ionization mode (EI) were recorded at 70 eV. The temperature of the ion source was 230°C, and the mass range was from 40 to 500 amu at full-scan mode. Peak identifications of the odorants were performed by comparison of mass spectra with those of the NIST 19.0 database (Agilent Technologies Inc., Santa Clara, CA, United States). Positive identification was achieved by comparison of their retention indices (RIs) and mass spectra with those of pure standards. The RIs of the odorants were calculated from the retention times of n-alkanes (C5–C30), according to a modified Kovats method (24).

To confirm the identification of these aroma compounds, the analytical procedures were also performed on a TG-5MS non-polar capillary column (5% phenyl methylpolysiloxane, 30 m × 0.25 mm × 0.25 μm, Thermo Fisher Scientific). The GC-MS analysis of extracts was implemented by a Thermo Trace 1300 gas chromatograph equipped with a Thermo ISQ LT mass selective detector system (Thermo Fisher Scientific). Helium (> 99.999%) was applied as the carrier gas at a flow rate of 1.0 ml/min. The temperature of the injector was set at 250°C. The oven temperature was programmed as follows: 40°C for 2 min, 1°C/min up to 50°C and held for 2 min, 3°C/min up to 70°C and held for 3 min, 6°C/min up to 230°C and held for 2 min, and 20°C/min up to 320°C and held for 4 min. All injections were set in split mode, and the split ratio was 30:1. The mass spectrometer was operated in the electron ionization mode with electron energy set as 70 eV and the mass range was from 43 to 500 amu at full-scan mode. The transfer line and ion source temperatures were both set to 300°C. Most aroma compounds were identified by comparing their retention indices (RIs) and mass spectra with those of pure standards.

#### Quantitative analysis

Quantitative analyses were routinely performed following the IS method, and the internal standard compounds were n-pentyl acetate, 4-octanol, 4-hydroxy-2-butanone, and 2-ethylbutyric acid. The selected ion monitoring (SIM) mode was adopted, and each analyte was quantified on the basis of the peak area using one quantitative fragment and two qualitative fragments (Table 1). The selected quantitative ions of the four IS were m/z 70, 69, 43, and 73, respectively. Moreover, standard calibration curves were used to quantify the target aroma compounds using a suitable capillary column based on the ratio of the peak area of the compound relative to the peak area of the internal standard to determine the concentration of the analyte.

**TABLE 1:** Aroma compounds identified by gas chromatography-mass spectrometry (GC-MS) in four grades of strong-aroma types of base Baijiu (base SAB).

No	Aroma compounds	^a^Identification	DB-WAX		TG-5MS		Monitored ions m/z
				
			RI	^b^LRI	RI	^b^LRI	
1	Ethyl acetate	MS, RI, S	881	884	614	613	43, 61, 70, 88
2	Diethyl acetal	MS, RI, S	906	900	726	725	45, 73, 103, 118
3	Ethyl butanoate	MS, RI, S	1035	1032	803	803	43, 71, 88, 116
4	2-butanol	MS, RI, S	1038	1041	604	598	45,59,74
5	1-propanol	MS, RI, S	1046	1049	539	532	59, 60
6	2-methylpropanol	MS, RI, S	1105	1094	624	622	43, 74
7	Ethyl pentanoate	MS, RI, S	1121	1120	903	900	57, 85, 101, 130
8	2-pentanol	MS, RI, S	1132	1124	701	706	45, 55, 73, 88
9	1-butanol	MS, RI, S	1156	1150	658	656	43, 56, 74
10	Ethyl 4-methylpentanoate	MS, RI, S	1180	1180	970	969	88, 99, 101, 144
11	2-methylbutanol	MS, RI, S	1206	1208	734	736	56, 70, 88
12	3-methylbutanol	MS, RI, S	1208	1211	732	730	43, 55, 88
13	Ethyl hexanoate	MS, RI, S	1218	1220	1009	1002	88, 99, 101, 144
14	1-pentanol	MS, RI, S	1246	1255	763	764	55, 70, 88
15	Hexyl acetate	MS, RI, S	1262	1265	1016	1014	56, 69, 84, 144
16	Propyl hexanoate	MS, RI, S	1305	1300	1097	1094	61, 99, 117, 158
17	Ethyl heptanoate	MS, RI, S	1314	1317	1100	1095	88, 101, 113, 158
18	Ethyl lactate	MS, RI, S	1337	1340	817	815	45, 75, 118
19	1-hexanol	MS, RI, S	1343	1345	868	867	55, 56, 69, 102
20	Butyl hexanoate	MS, RI, S	1396	1392	1193	1188	56, 99, 117, 172
21	Ethyl octanoate	MS, RI, S	1419	1420	1199	1195	88, 101, 127, 172
22	Isopentyl hexanoate	MS, RI, S	1438	1450	1253	1250	70, 71, 99, 186
23	Acetic acid	MS, RI, S	1442	1441	620	625	43, 45, 60
24	1-octanol	MS, RI, S	1542	1546	1079	1070	56, 70, 84, 130
25	2-methylpropanoic acid	MS, RI, S	1556	1564	789	785	43, 73, 88
26	2,3-butanediol	MS, RI, S	1567	1576	−	−	45, 57, 90
27	Hexyl hexanoate	MS, RI, S	1593	1593	1386	1385	84, 99, 117, 200
28	Butanoic acid	MS, RI, S	1615	1610	793	793	45, 60, 73, 88
29	Ethyl decanoate	MS, RI, S	1622	1629	1395	1392	88, 101, 155, 200
30	Isoamyl octanoate	MS, RI, S	1643	1651	1447	1450	70, 127, 145, 214
31	Ethyl benzoate	MS, RI, S	1655	1652	1172	1170	105, 122, 150
32	3-methylbutanoic acid	MS, RI, S	1658	1660	864	866	60, 87, 102
33	Diethyl butanedioate	MS, RI, S	1666	1667	1185	1181	101, 128, 129, 174
34	Pentanoic acid	MS, RI, S	1726	1729	−	−	60, 73, 102
35	Ethyl phenylacetate	MS, RI, S	1775	1785	1247	1252	65, 91, 164
36	4-methylpentanoic acid	MS, RI, S	1791	1792	−	−	73, 74, 83, 116
37	Hexyl octanoate	MS, RI, S	1795	1795	1583	1582	84, 127, 145, 228
38	Ethyl dodecanoate	MS, RI, S	1829	1828	1595	1597	88, 101, 183, 228
39	Hexanoic acid	MS, RI, S	1833	1827	−	−	60, 73, 87, 116
40	ethyl 3-phenylpropionate	MS, RI, S	1874	1872	1350	1350	91, 104, 178
41	Phenylethyl alcohol	MS, RI, S	1903	1901	1112	1116	91, 92, 122
42	Heptanoic acid	MS, RI, S	1940	1943	1070	1071	60, 73, 87, 130
43	4-methylguaiacol	MS, RI, S	1951	1956	1192	1192	95, 123, 138
44	4-ethylguaiacol	MS, RI, S	2023	2032	1280	1280	122, 137, 152
45	Ethyl tetradecanoate	MS, RI, S	2034	2040	1794	1793	88, 101, 256
46	Octanoic acid	MS, RI, S	2045	2050	1190	1191	60, 73, 101, 144
47	4-methylphenol	MS, RI, S	2075	2079	1082	1084	77, 107, 108
48	Ethyl hexadecanoate	MS, RI, S	2240	2246	1995	1994	88, 101, 284
49	Ethyl octadecanoate	MS, RI, S	2441	2455	2194	2194	88, 101, 312
50	Ethyl oleate	MS, RI, S	2461	2461	2168	2169	68, 88, 264, 310
51	Ethyl linoleate	MS, RI, S	2508	2510	2163	2163	81, 95, 109, 308
52	Ethyl linolenate	MS, RI, S	2575	2578	2169	2173	79, 95, 108, 306

^a^MS, compounds were identified by MS spectra; RI, the retention index of compounds were identified on FFAP and TG-5MS by comparison to reference standards; S, compounds were identified by standards.

^b^LRI, Literature RI.

### Statistical analysis

All chemical analyses in this work were carried out in triplicate, and the concentrations of each aroma compound acquired from GC-MS analysis were expressed as the means ± standard deviation (SD). The raw data obtained from the Smartongue were analyzed by both PCA and DFA pattern recognition techniques. PCA is mainly used to model, compress, and visualize multivariate data by setting a new coordinate system in which Euclidean distances between the objects remain the same. As a well-known unsupervised method, PCA allows the reduction of multidimensional data and simplifies the interpretation of the data by a few principal components (25). DFA is used to examine differences between or among groups by using a discriminant prediction equation, which allows for the rejection of variables that are little related to group distinctions (26). Among them, 120 types of base SAB samples were used to establish a quality grading model through PCA and DFA by the Smartongue system version 3.0 as a calibration set. The rest 20 base SAB samples were used to verify these models as validation set.

## Results and discussion

### Sensory evaluation

A total of 140 base SAB samples were classified into 4 grades (A, B, C, and D) through their taste characteristics by the eight sommeliers, and 35 types of base SAB samples of each grade were included. Among the sensory evaluation of these four grades, the base SAB from grades A, B, and C all showed strong cellar fragrance, pure, long aftertaste, and no peculiar smell with a tendency to decrease; however, grade D showed fermented grains flavor, less cellar fragrance, and short aftertaste. These results suggest that the rank of four grades from good to bad were successively A, B, C, and D according to the sensory requirements of national standard of GB/T 10781.1-2021 (27).

### Smartongue analysis

**FIGURE 1:**
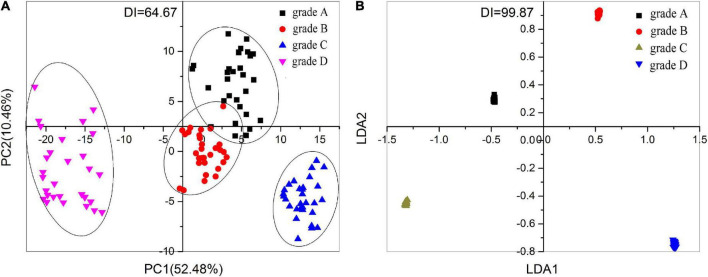
Multivariate statistical analysis based on the result of the Smartongue analysis: loading plot of **(A)** principal component analysis (PCA) and **(B)** discriminant function analysis (DFA) for the classification of 120 strong-aroma types of base Baijiu (base SAB) samples from four different grades.

In this study, 120 types of base SAB samples of 4 different grades (30 samples of each grade) were tested by the Smartongue and the map of its PCA and DFA are shown in Figure 1. As shown in Figure 1A, base SAB samples of grade C and grade D could be classified apparently by PCA; however, base SAB from grade A and grade B can be only discriminated with a little overlap. Moreover, Figure 1A also shows the score plot relative to the first and second principal components (PC1 and PC2) were 52.48 and 10.46%, respectively. The total principal component score (62.94%) indicated that base SAB samples could only be discriminated by PCA roughly. The discrimination index (DI, a number to evaluate the separation level for the above non-linear multivariate data analysis methods and its maximum DI value is 100%, indicating the best separation of the samples) value of PCA is 64.67, which also means base SAB samples could be discriminated reluctantly. As can be observed in Figure 1B, the four grades base SAB samples of A, B, C, and D could be discriminated obviously by the DFA. It could be seen that DFA had a good separation ability among these four grades (A, B, C, and D) base SAB samples with a DI value of 99.87. The analysis above showed that DFA was more applicable to grade base SAB samples than PCA. Therefore, the DFA was used to establish a quality grading prediction model.

**FIGURE 2:**
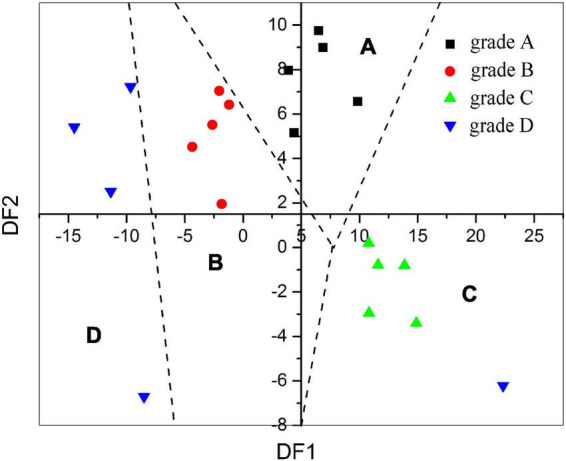
The established DFA model map was verified by 20 real SAB samples.

On the basis of the DFA prediction model, the predicted quality grade result of the validation set is shown in Figure 2. As shown in Figure 2, 20 validations set base SAB samples from four grades could be classified obviously by the DFA model except for one base SAB sample from grade D. Hence, all the results above revealed that the Smartongue system was capable of classifying the base SAB and quality grade predicting results with an average accuracy up to 95%.

### Qualitative analysis

A total of 52 aroma compounds, including 27 esters, 12 alcohols, 9 acids, 3 phenols, and 1 acetal, were determined in base SAB samples by direct injection and liquid-liquid extraction (LLE) coupled with GC-MS analysis (Table 1). As exhibited in Table 1, all aroma compounds could be successfully identified in grades A, B, and C base SAB samples while only 38 aroma compounds were identified in grade D, which suggested that the component is more abundant in higher grade samples. Main volatile compounds of SAB, such as ethyl acetate, ethyl lactate, ethyl butanoate, ethyl hexanoate, 1-butanol, 3-methylbutanol, butanoic acid, and hexanoic acid have been determined in previous literature (3, 28). Among them, ethyl hexanoate, hexyl hexanoate, ethyl pentanoate, and hexanoic acid were regarded as odor-active compounds and contribute greatly to the odors of SAB (29). Besides, most of these compounds have been earlier identified as aroma compounds in other aroma types of Baijiu (30). Hence, the qualitative method used in this study turned out to be appropriate and reliable.

### Quantitative analysis

To gain a deeper insight into the characterization and relevance of base SAB samples, a total of 52 aroma compounds were quantitated in these four grades. As shown in Table 2, the obtained standard curves were observed to have a good linearity with a correlation coefficient (*R*^2^) ≥ 0.99 and the RSDs in triplicate of samples were ≤ 10%, which illustrated the good precision of the quantitative methods. Based on these result, ethyl hexanoate were present in the highest concentrations in grades A, B, C, and D (2,712.98, 2,245.57, 2,047.17, and 288.41 mg/L, respectively), followed by ethyl acetate (1,556.69 mg/L-A, 1,167.78 mg/L-B, 1,000.94 mg/L-C, and 979.83 mg/L-D), ethyl lactate (1,038.02 mg/L-A, 1,457.65 mg/L-B, 1,542.16 mg/L-C, and 1,570.86 mg/L-D), and ethyl butanoate (269.31 mg/L-A, 226.02 mg/L-B, 200.45 mg/L-C, and 107.12 mg/L-D). These 4 compounds were all present at levels above 100 mg/L, and they were taken as the key volatile components of SAB. Additionally, hexanoic acid (909.14 mg/L-A, 808.67 mg/L-B, 490.83 mg/L-C, and 59.62 mg/L-D), acetic acid (778.56 mg/L-A, 649.30 mg/L-B, 491.79 mg/L-C, and 528.11 mg/L-D), 1-propanol (596.94 mg/L-A, 320.97 mg/L-B, 238.34 mg/L-C, and 183.97 mg/L-D), and diethyl acetal (513.49 mg/L-A, 434.37 mg/L-B, 361.09 mg/L-C, and 250.17 mg/L-D) were also presented in high concentrations. Moreover, as shown in Table 3, the content of ethyl hexanoate, total acid, and total ester in grade A had reached the first grade standard of high alcohol content Chinese strong flavor Baijiu following the national standard of GB/T 10781.1-2021, but partly achieved in grades B, C, and D. These findings indicated that a high-grade base SAB sample (e.g., grade A) was superior to a low-grade base SAB sample (e.g., grade D). Besides, most of the concentrations of aroma compounds in grades A, B, C, and D presented a sequentially decreasing trend. Overall, the aforementioned results demonstrated that the quality ranks of four grades of base SAB samples from good to bad were A, B, C, and D, successively, which is consistent with the grading result acquired from the electronic tongue and human sensory evaluations.

**TABLE 2:** Standard curves and concentrations of 52 aroma compounds in four grades of base SAB.

No	Aroma compound	Standard curve			A		B		C		D	
						
		Slope	Intercept	R^2^	^a^av ± SD(mg/L)	^b^RSD(%)	av ± SD (mg/L)	RSD (%)	av ± SD (mg/L)	RSD (%)	av ± SD (mg/L)	RSD (%)
13	Ethyl hexanoate	31.302	−2.048	0.9994	2712.98 ± 21.16	0.78	2245.57 ± 11.00	0.49	2047.17 ± 7.98	0.39	288.41 ± 6.81	2.36
1	Ethyl acetate	307.67	−1.382	0.9957	1556.69 ± 69.90	4.49	1167.78 ± 6.66	0.57	1000.94 ± 16.92	1.69	979.83 ± 15.58	1.59
18	Ethyl lactate	41.556	−1.2471	0.9991	1038.02 ± 11.31	1.09	1457.65 ± 2.19	0.15	1542.16 ± 4.78	0.31	1570.86 ± 24.51	1.56
39	Hexanoic acid	22.832	−0.0237	0.9996	909.14 ± 4.18	0.46	808.67 ± 19.25	2.38	490.83 ± 1.23	0.25	59.62 ± 1.08	1.81
23	Acetic acid	161.78	−0.912	0.9945	778.56 ± 14.87	1.91	649.30 ± 7.34	1.13	491.79 ± 17.61	3.58	528.11 ± 12.67	2.40
5	1-propanol	76.956	−0.9049	0.9992	596.94 ± 12.37	2.07	320.97 ± 4.01	1.25	238.34 ± 0.60	0.25	183.97 ± 4.10	2.23
2	Diethyl acetal	78.695	−0.7763	0.9976	513.49 ± 18.13	3.53	434.37 ± 2.91	0.67	361.09 ± 5.81	1.61	250.17 ± 3.40	1.36
12	3-methylbutanol	96.038	−1.0605	0.9993	288.68 ± 4.85	1.68	239.98 ± 0.05	0.02	278.47 ± 28.49	10.23	23.37 ± 0.50	2.15
3	Ethyl butanoate	81.061	−1.6081	0.9994	269.31 ± 7.57	2.81	226.02 ± 1.83	0.81	200.45 ± 2.16	1.08	107.12 ± 4.17	3.89
28	Butanoic acid	18.57	1.8407	0.9965	267.51 ± 5.11	1.91	238.30 ± 2.91	1.22	151.17 ± 0.33	0.22	46.88 ± 2.97	6.34
6	2-methylpropanol	42.383	−1.6759	0.9995	173.46 ± 3.02	1.74	168.29 ± 1.06	0.63	172.85 ± 0.02	0.01	126.89 ± 3.45	2.72
21	Ethyl octanoate	40.712	−3.3363	0.9941	165.24 ± 0.78	0.47	168.38 ± 1.40	0.83	182.95 ± 0.20	0.11	7.00 ± 0.22	3.13
4	2-butanol	202.58	−0.873	0.9996	165.17 ± 2.68	1.62	28.18 ± 0.48	1.69	20.91 ± 0.16	0.77	13.33 ± 0.09	0.65
9	1-butanol	93.41	−0.9313	0.9993	163.19 ± 0.39	0.24	143.04 ± 5.38	3.76	179.70 ± 2.61	1.45	152.63 ± 3.04	1.99
11	2-methylbutanol	81.863	−1.1663	0.9993	119.46 ± 1.04	0.87	107.30 ± 0.26	0.24	112.69 ± 0.36	0.32	74.35 ± 1.10	1.48
19	1-hexanol	36.08	−0.9008	0.9992	96.74 ± 0.52	0.54	110.04 ± 0.57	0.52	84.21 ± 0.08	0.09	25.30 ± 1.17	4.61
7	Ethyl pentanoate	30.692	−1.6759	0.9994	57.18 ± 1.36	2.37	39.24 ± 0.27	0.68	40.16 ± 0.17	0.43	10.25 ± 0.48	4.65
17	Ethyl heptanoate	25.251	−1.8775	0.9995	56.53 ± 0.03	0.05	51.13 ± 0.21	0.42	58.86 ± 0.21	0.36	2.20 ± 0.01	0.31
48	Ethyl hexadecanoate	12.978	−0.7642	0.9994	40.91 ± 0.36	0.87	35.61 ± 0.32	0.91	41.04 ± 0.02	0.05	7.91 ± 0.15	1.85
8	2-pentanol	25.355	−1.3041	0.9992	40.83 ± 0.61	1.49	9.18 ± 0.75	8.16	6.13 ± 0.06	1.03	1.76 ± 0.06	3.66
34	pentanoic acid	18.131	1.6381	0.9963	39.89 ± 0.71	1.79	25.65 ± 0.44	1.70	18.19 ± 0.002	0.01	3.86 ± 0.14	3.66
25	2-methylpropanoic acid	22.565	0.9897	0.9965	34.45 ± 0.54	1.58	35.52 ± 0.32	0.91	31.74 ± 0.13	0.42	32.38 ± 2.92	9.02
46	Octanoic acid	26.695	1.127	0.9981	27.72 ± 0.05	0.19	39.17 ± 0.02	0.05	18.36 ± 0.06	0.34	^c^nd	
22	Isopentyl hexanoate	40.267	−3.0078	0.9934	26.20 ± 0.05	0.19	21.25 ± 0.32	1.52	18.79 ± 0.38	2.00	nd	
32	3-methylbutanoic acid	28.585	1.4284	0.997	22.83 ± 0.47	2.04	21.49 ± 0.31	1.45	12.97 ± 0.01	0.06	4.75 ± 0.14	2.85
51	Ethyl linoleate	22.675	−0.4406	0.9996	21.85 ± 0.04	0.18	23.22 ± 0.19	0.80	22.09 ± 0.10	0.47	29.06 ± 0.55	1.89
27	Hexyl hexanoate	11.53	−0.0939	0.9973	21.31 ± 0.80	3.75	22.55 ± 0.07	0.32	12.91 ± 0.13	1.04	0.21 ± 0.01	3.12
40	Ethyl 3-phenylpropionate	11.602	−0.6449	0.9993	18.84 ± 0.16	0.85	16.29 ± 0.14	0.86	7.91 ± 0.03	0.43	1.62 ± 0.05	3.09
50	Ethyl oleate	42.673	−0.2761	0.9997	15.17 ± 0.09	0.57	16.54 ± 0.11	0.64	17.79 ± 0.09	0.50	22.94 ± 0.05	0.22
42	Heptanoic acid	21.417	1.9262	0.9988	13.92 ± 0.02	0.12	13.97 ± 0.16	1.13	8.06 ± 0.03	0.34	nd	
14	1-pentanol	39.533	−1.5388	0.9993	13.64 ± 0.09	0.64	11.21 ± 0.73	6.49	11.21 ± 0.02	0.16	3.90 ± 0.11	2.84
16	Propyl hexanoate	26.416	−1.5796	0.9992	13.01 ± 0.001	0.01	6.69 ± 0.06	0.95	3.03 ± 0.02	0.55	nd	
20	Butyl hexanoate	20.406	−3.014	0.9932	12.27 ± 0.006	0.05	13.81 ± 0.14	1.01	10.93 ± 0.02	0.17	nd	
29	Ethyl decanoate	9.6506	−0.0487	0.9974	7.75 ± 0.73	9.43	7.74 ± 0.08	0.97	7.24 ± 0.08	1.10	3.50 ± 0.24	6.98
33	Eiethyl butanedioate	8.6862	−0.3331	0.9972	7.07 ± 0.20	2.84	14.49 ± 0.21	1.44	9.36 ± 0.04	0.43	25.74 ± 2.33	9.04
10	Ethyl 4-methylpentanoate	27.917	−1.9502	0.9994	4.20 ± 0.08	1.83	3.42 ± 0.01	0.33	1.23 ± 0.02	1.53	nd	
26	2,3-butanediol	24.525	0.5784	0.9968	4.15 ± 0.10	2.49	6.48 ± 0.05	0.79	8.51 ± 0.11	1.33	8.24 ± 0.54	6.58
41	Phenylethyl alcohol	9.2004	−0.5347	0.9993	3.48 ± 3.0 × 10^–4^	0.01	3.50 ± 0.04	1.04	3.94 ± 0.01	0.16	5.65 ± 0.47	8.31
36	4-methylpentanoic acid	37.98	1.4439	0.9989	3.14 ± 0.01	0.47	3.09 ± 0.01	0.32	1.85 ± 0.01	0.37	nd	
47	4-methylphenol	10.291	−0.5812	0.9992	2.61 ± 0.01	0.34	2.83 ± 0.02	0.74	1.10 ± 7.70 × 10^–4^	0.07	nd	
49	Ethyl octadecanoate	36.649	−0.5657	0.9995	2.09 ± 0.02	1.15	1.82 ± 0.18	9.66	1.80 ± 0.01	0.49	nd	
52	Ethyl linolenate	46.229	0.0968	0.9998	2.08 ± 0.01	0.43	2.46 ± 3.69 × 10^–3^	0.15	2.18 ± 0.01	0.60	2.92 ± 0.13	4.43
45	Ethyl tetradecanoate	13.849	−0.5317	0.9994	2.03 ± 0.04	1.91	1.93 ± 0.01	0.62	2.11 ± 0.01	0.27	1.75 ± 0.05	2.85
35	Ethyl phenylacetate	9.0471	−0.585	0.9993	1.90 ± 0.02	1.10	1.93 ± 0.02	0.97	3.07 ± 0.02	0.71	0.79 ± 0.03	4.00
15	Hexyl acetate	35.685	−1.4155	0.9992	1.79 ± 0.04	1.96	2.30 ± 0.02	0.70	0.56 ± 2.74 × 10^–3^	0.49	nd	
31	Ethyl benzoate	11.114	−0.6108	0.9975	1.71 ± 0.08	4.52	0.54 ± 0.02	2.94	0.48 ± 0.01	1.66	nd	
43	4-methylguaiacol	11.703	−0.5721	0.9993	1.66 ± 0.04	2.59	2.68 ± 5.36 × 10^–4^	0.02	1.37 ± 2.74 × 10^–3^	0.20	1.42 ± 0.03	2.13
38	Ethyl dodecanoate	11.096	−0.097	0.9988	1.63 ± 0.04	2.32	1.32 ± 0.01	0.79	1.54 ± 0.01	0.42	0.89 ± 0.01	1.09
24	1-octanol	22.072	0.3593	0.9977	1.45 ± 0.03	2.26	2.42 ± 0.02	0.67	1.46 ± 0.01	0.39	nd	
44	4-ethylguaiacol	9.3486	−0.2911	0.9991	1.38 ± 0.01	0.74	1.5 ± 3.00 × 10^–3^	0.20	0.87 ± 8.70 × 10^–4^	0.10	1.74 ± 0.03	1.71
30	Isoamyl octanoate	10.48	−0.0887	0.9975	0.67 ± 0.03	4.73	0.64 ± 0.01	1.38	0.61 ± 0.01	1.01	nd	
37	Hexyl octanoate	13.831	−0.507	0.9993	0.55 ± 0.001	0.23	1.16 ± 0.01	0.82	0.32 ± 3.52 × 10^–3^	1.10	nd	

^a^av ± SD (n = 3), average concentration of triplicates; ^b^RSD, relative standard deviation of the average concentration; ^c^nd, not detected.

**TABLE 3:** Physicochemical indexes comparison of 4 grades of base SAB samples.

Item	^a^First grade	A	B	C	D
Total acid (of acetic acid count)/(g/L)	≥ 0.30	✓	✓	✓	✓
Total ester (of ethyl acetate count) /(g/L)	≥ 1.50	✓	✘	✘	✘
Ethyl hexanoate/(g/L)	0.60–2.50	✓	✓	✓	✘

^a^First grade, the physicochemical standard of SAB with high alcohol content (41–68% ABV) following the national standard of GB/T 10781.1-2021.

## Conclusion

In the present study, an electronic tongue combined with the GC-MS method was developed to grade high-alcoholic base SAB for the first time. The E-tongue showed a good prediction in different grades of base SAB when models were established using DFA, which suggests that the E-tongue combined with data modeling is promising for flavor quantification and quality grading. Moreover, to gain a deeper insight into the characterization and relevance of base SAB samples, a total of 52 aroma compounds were identified in four grades of base SAB and differences in the composition of volatile components from four grades were observed by GC-MS analysis. In general, the variety and concentration of high-grade base SAB were more than that of low grade, which showed a good agreement with human sensory evaluation results. These findings provide a guide for Baijiu industries to select the proper method to the overall quality grading for base Baijiu. Nonetheless, we still question the applicability of E-tongue in quantifying the overall quality of base Baijiu from other aroma types. Further research on the quality classification by using more base Baijiu varieties will confirm and improve our findings.

## Data availability statement

The original contributions presented in this study are included in the article/supplementary material, further inquiries can be directed to the corresponding authors.

## Author contributions

LA: investigation, formal analysis, and writing—original draft. KG: methodology, validation, data curation, and writing—original draft. XD: investigation and visualization. WD: writing—review and editing and funding acquisition. XS: data curation and writing—review and editing. BS: editing, supervision, and funding. JS: writing—review and editing and visualization. GL: methodology and validation. AL: supervision and funding. HL: methodology and software. FZ: supervision and validation. All authors have read and approved the submitted version.
